# The Temporal and Spatial Responses of Large and Medium Mammals to Anthropogenic Disturbances in Montane Southwest China

**DOI:** 10.1002/ece3.72024

**Published:** 2025-08-29

**Authors:** Qiuxian Li, Xinyu Cui, Qingsong Jiang, Hangshu Xiao, Assan Meshach, Huaqiang Zhou, Yin Li, Zejun Zhang, Mingsheng Hong

**Affiliations:** ^1^ Liziping Giant Panda's Ecology and Conservation Observation and Research Station of Sichuan Province (Science and Technology Department of Sichuan Province) China West Normal University Nanchong China; ^2^ Key Laboratory of Southwest China Wildlife Resources Conservation (Ministry of Education) China West Normal University Nanchong China; ^3^ College of Pharmacy Chengdu University of Traditional Chinese Medicine Chengdu China

**Keywords:** anthropogenic disturbances, camera traps, daily activity rhythm, Kazila mountains, relative abundance index (RAI), spatial niches

## Abstract

Humans, as super predators, influence wildlife behavior through both direct predation and indirect fear effects, prompting spatial and temporal adaptations. In landscapes where human–wildlife coexistence is prevalent, understanding the spatiotemporal strategies employed by rare wildlife in response to anthropogenic disturbance is essential for effective biodiversity conservation. From July 2019 to September 2024, we deployed 62 camera traps in the Kazila Mountain region of Yajiang County, Sichuan Province, resulting in 6204 independent detections of rare wildlife and 722 recorded human activity events. Using occupancy modeling and kernel density estimation, we evaluated the influence of human presence and environmental variables on the behavior of forest‐dwelling wildlife in the mountainous areas of southwestern China. Our camera traps recorded six herbivore species (e.g., tufted deer, sambar), nine carnivore species (e.g., red fox, leopard), and five omnivore species (e.g., wild boar, rhesus macaque). Representative species from each trophic group were selected for detailed analysis based on detection frequency. Temporally, all three groups exhibited distinct diel activity peaks that differed significantly from those of human activity. Carnivores (leopard, red fox) and herbivores (sambar, tufted deer) altered their activity rhythms in response to human presence, while omnivores (wild boar, rhesus macaque) showed substantial overlap with human activity periods but avoided peak disturbance times. Spatially, carnivores tended to use areas with greater human and livestock presence, whereas herbivores preferred locations further from roads and settlements, typically in gentler terrain. Among omnivores, rhesus macaques avoided areas with high human and livestock activity, while wild boars appeared largely unaffected by such disturbances. These findings offer important insights into the conservation of rare wildlife in the mountainous regions of southwest China. This study underscores the utility of camera traps in directly monitoring human disturbance and quantifying its ecological impacts. The differential spatiotemporal responses observed among threatened medium‐ and large‐sized mammals highlight their behavioral plasticity in disturbed environments, aiding predictions of species' responses to future environmental change based on current adaptation strategies.

## Graphical abstract

This study provides new insights for conserving rare wildlife in southwest China's mountainous regions, highlighting the importance of using camera traps to directly monitor anthropogenic disturbance and quantify its impact on species survival. The varying responses of threatened medium and large mammals to human activities emphasize the spatiotemporal behavioral plasticity of animals in disturbed environments, helping predict how species might respond to future environmental changes based on their current spatiotemporal strategies.

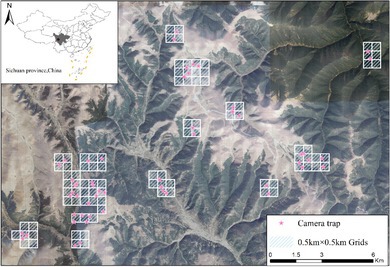

## 1 Introduction

Human impacts on natural ecosystems have become nearly ubiquitous, with at least 95% of the global land area experiencing some degree of anthropogenic modification (Kennedy et al. 2019). These human activities have significantly altered wildlife habitats, contributing to global biodiversity loss (Martínez‐Ramos et al. 2016; Dirzo et al. 2014) and disrupting essential ecosystem processes and functions (Tobler, Hartley, et al. 2015). As human expansion continues, wildlife habitats face increasing fragmentation, degradation, and loss (Côté et al. 2016), which negatively affects species distributions, activity patterns, and reproductive success (Tobler, Zuniga, et al. 2015). In response, the ecological consequences of anthropogenic disturbance have become a major focus in wildlife conservation and behavioral ecology (Hu et al. 2022). Although numerous studies have documented the negative impacts of human activity on biodiversity (Dirzo et al. 2014), comparative investigations of how different types of human disturbances affect species' activity patterns and habitat preferences remain limited (Boron et al. 2019; Li, Hu, et al. 2022).

Wildlife often adapts to human‐modified environments by shifting their activity patterns or altering their spatial behavior to reduce encounters with humans (Laméris et al. 2019). For example, some carnivores may perceive human presence as a predation threat and avoid areas or times with high human activity (Darimont et al. 2015). Overall mammalian species richness tends to decline in anthropogenic landscapes (Li, Hu, et al. 2022), and many species respond by exhibiting spatial overlap with humans while adjusting their temporal activity to avoid direct contact (Watabe and Saito 2021). In particular, increased nocturnality in response to human disturbance is a widely documented strategy across taxa, regions, and habitat types (Gaynor et al. 2018). According to predator–prey theory, short‐term and predictable disturbances—such as grazing or daily human activity—may lead to temporal avoidance behaviors (Nickel et al. 2020); whereas prolonged and widespread disturbances—such as urbanization or infrastructure development—may cause more profound changes in habitat selection (Tucker et al. 2018). These behavioral adaptations are also species‐specific, depending on the type and intensity of the disturbance. Large carnivores, for instance, typically reduce their presence in heavily disturbed areas, relying more on temporal avoidance (Nickel et al. 2020), while smaller carnivores may tolerate moderately disturbed habitats but reduce activity in areas with high human presence (Li, Hu, et al. 2022).

Camera trap technology has become an essential tool for studying wildlife in remote or rugged environments and for collecting long‐term, noninvasive data on animal abundance, distribution, and behavior (Rovero and Zimmermann 2016; Li, Zhang, et al. 2024). These devices allow for simultaneous monitoring of both wildlife and human activities, enabling researchers to quantify spatiotemporal overlap and behavioral adaptations. Data from camera traps have been widely used to explore interspecific interactions and evaluate the effects of human disturbance on wildlife populations (Frey et al. 2017). For example, Gaynor et al. (2018) synthesized 76 studies and found consistent increases in nocturnality among wildlife in response to human activity, regardless of geographic or ecological context. Spatially, species richness and diversity are often reduced in the vicinity of anthropogenic structures or settlements (Kitchen et al. 2000). As such, camera trap data provide a robust foundation for investigating how wildlife respond to human presence across both temporal and spatial dimensions.

In this study, we used camera trap data from the Kazila Mountain area in Yajiang County, Sichuan Province, to investigate the spatiotemporal responses of medium and large mammals to anthropogenic disturbance in a mountainous landscape of southwestern China. By applying species occupancy models and kernel density estimation, we analyzed how human activity influences species distributions and diel activity patterns across different trophic groups. Therefore, the objectives of this study were to: (1) investigate activity patterns of medium and large mammals in the study area; (2) examine differences in activity patterns between low and high disturbance areas; and (3) determine occupancy patterns of medium and large mammals and their influencing factors.

## 2 Materials and Methods

### 2.1 Study Area

This study was conducted in the Kazila Mountain region of southwestern China. Kazila Mountain is located in Eluo Village, Xieluo Town, Yajiang County, Ganzi Prefecture, Sichuan Province. The study area is situated in the southeastern edge of the Qinghai–Tibet Plateau, in the middle section of the Hengduan Mountains, within the plateau region between the Daxue Mountains and Shaluli Mountains. The average elevation is approximately 3800 m, with an average annual precipitation of about 705 mm. The rainy season is concentrated from May to September each year (Tie et al. 2021). From November to April of the following year, the area is mostly covered by snow (Li, Zhang, et al. 2024). The Eluo River, a secondary tributary of the Yalong River, flows from north to south along the eastern side of Kazila Mountain. Human activities such as grazing and mushroom harvesting are common in the study area, which is also home to various rare wildlife species, including leopards, lynx, alpine musk deer, and forest musk deer (Hui et al. 2016).

### 2.2 Experimental Design

**FIGURE 1:**
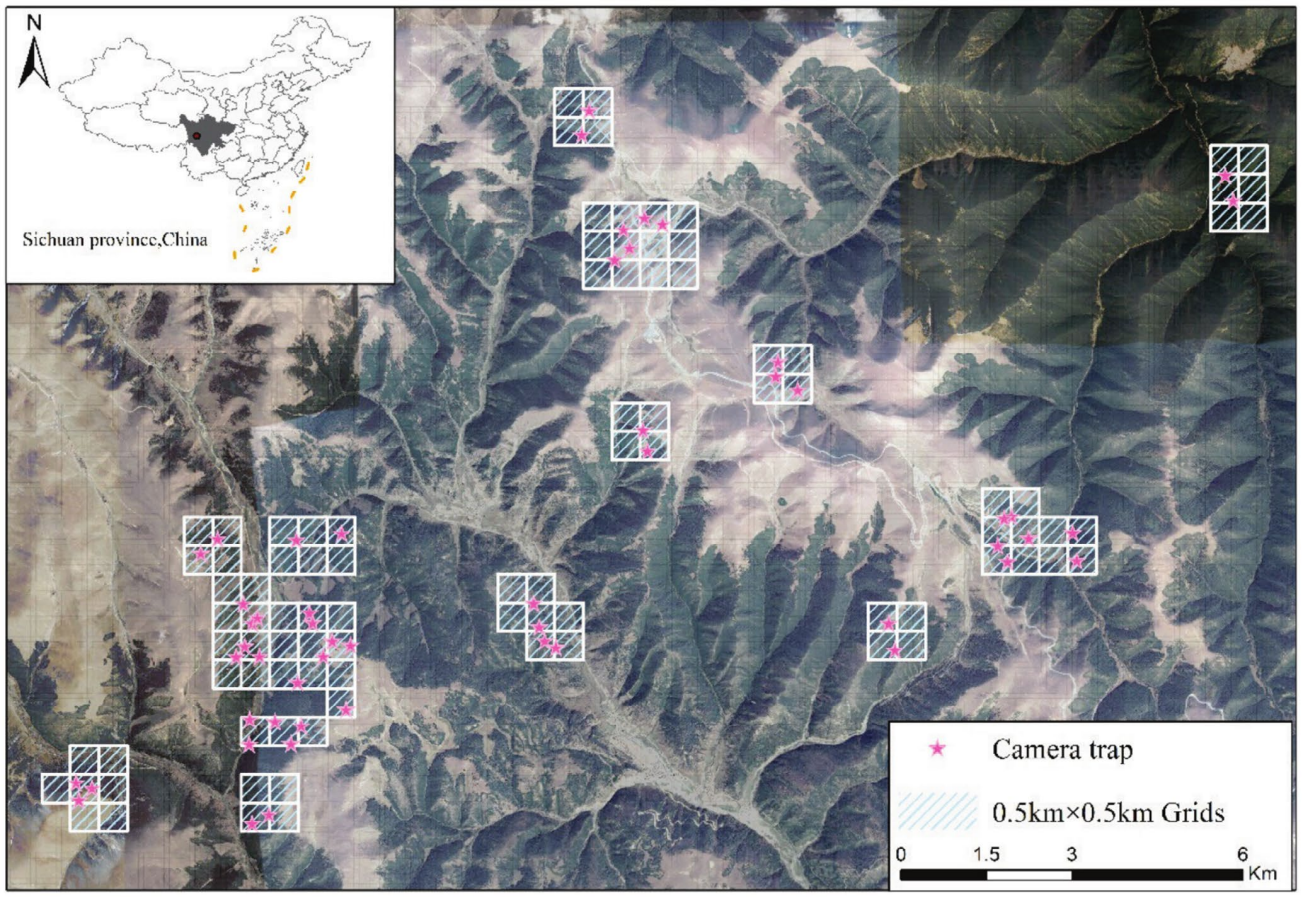
Camera trap locations in the Kazila Mountain area, Sichuan, China. The inset shows the study area's location in western China. Dark areas represent dense vegetation, while light areas indicate sparse vegetation. Roads and villages are primarily located in valleys or open areas, with elevations ranging from approximately 3100–4300 m. Map downloaded from Google Earth (https://earth.google.com/).

From July 2019 to September 2024, we deployed 62 noninvasive infrared cameras (model LTI‐6511, Shenzhen, China) in the Kazila Mountain region to monitor medium and large mammals. We established a 500 m × 500 m grid across the study area. Based on interviews, transect surveys, and accessibility considerations, we placed cameras in locations with high animal activity probability, including forest clearings, ridges, animal trails, and near water sources (Cong et al. 2024). The infrared cameras were programmed to record the time and date on a 24‐h clock when triggered, capturing three photographs and a 10‐s video per trigger event, with a minimum interval of 10 s between consecutive triggers. The total operational days for each camera were calculated from deployment to the last recorded photograph or video. We conducted annual maintenance from April to June, downloading data and replacing batteries (Kays et al. 2020). Cameras were mounted on trees at heights of 40–80 cm above ground, covering various habitats including mixed coniferous‐broadleaf forests, boreal coniferous forests, alpine shrubs, and alpine meadows. The minimum distance between camera stations was 250 m (Wang et al. 2018; Tian et al. 2022) (Figure 1).

### 2.3 Data Analysis

#### 2.3.1 Relative Abundance Index

We recorded all medium and large wild mammals and various human disturbances captured by the cameras. We defined all photographs or videos of the same species captured within a 30‐min window as a single independent detection. The relative abundance index (RAI) was calculated using the following formula: RAI = (independent detections/total camera days) × 1000 (Chen et al. 2024).

#### 2.3.2 Selection of Dominant Species

We focused our analysis on medium‐ and large‐bodied mammals with body mass > 500 g, as they have larger space and dietary requirements, high sensitivity to human activities, and considerable behavioral plasticity (Gaynor et al. 2018; Li, Hu, et al. 2022). We selected species by trophic guild to compare responses to anthropogenic activities across different food chain levels, including carnivores, herbivores, and omnivores. Species consuming ≥ 90% animal‐based diet were categorized as carnivores, those consuming ≥ 90% plant‐based diet as herbivores, and species consuming < 90% of either animal‐ or plant‐based diet as omnivores (Atwood et al. 2020). Using the EltonTraits database combined with species activity patterns (Mazel et al. 2017; Wilman et al. 2014), we selected rare wildlife species from different trophic guilds for analysis.

#### 2.3.3 Temporal Analysis

The temporal niche overlap index, ranging from 0 (no overlap) to 1 (complete overlap), was used to assess temporal overlap among species under anthropogenic disturbance. The overlap index (Δ) was estimated using nonparametric kernel density estimation and temporal data from camera trap observations. Δ4 was used for analyses with sample sizes > 75, while Δ1 was used for smaller sample sizes (Romero‐Muñoz et al. 2010; Li, Zhang, et al. 2024). Overlap coefficients were categorized as: Δ < 0.5 (low overlap), 0.5 ≤ Δ < 0.7 (moderate overlap), and Δ ≥ 0.7 (high overlap) (Blanco et al. 2017). We generated 10,000 bootstrap samples with 95% confidence intervals to test the reliability of overlap values (Ridout and Linkie 2009). Using the mean relative abundance of humans and livestock as a threshold, we classified each camera trap site as having high or low human activity. We then analyzed differences in dominant species between high and low human activity areas (Oberosler et al. 2017). All analyses were conducted using the “overlap” package in R 4.3.2, with the “compareCkern” function from the “activity” package used to calculate differences in daily activity patterns (Ridout and Linkie 2009; Rowcliffe et al. 2014).

#### 2.3.4 Spatial Analysis

To investigate the effects of anthropogenic disturbance and environmental factors on the spatial distribution of medium‐ and large‐bodied mammals in the study area, we conducted single‐season, single‐species occupancy models to estimate occupancy rates (*ψ*) and detection probabilities (*p*) for representative species (Moreno‐Sosa et al. 2022; MacKenzie et al. 2017; Li, Li, et al. 2022). To ensure independence of captures, we only selected photos and videos of the same species that were separated by at least 30 min for analysis. We created a matrix using data from the most recent year, recording detections of representative species at each camera location within 30‐day intervals (0–30, 31–60, 61–90, 91–120, 121–150, 151–180, 181–210, 211–240, 241–270, 271–300, 301–330, > 330 days) (Cong et al. 2024; Li, Xue, et al. 2022).

Based on previous studies on habitat use and anthropogenic disturbance of medium‐ and large‐bodied animals, we selected five environmental variables and three anthropogenic disturbance factors for model construction (Alexander et al. 2016; Gorczynski et al. 2022). Topographic variables, including elevation (ELE), slope (SLP), and aspect (ASP), are fundamental elements of wildlife habitat and are closely related to other environmental factors (Chen et al. 2024). Vegetation variables included the enhanced vegetation index (EVI), which provides information on habitat concealment. Environmental variables included distance to water sources (DTW). Disturbance variables included distance to roads (DTR), distance to residential points (DTS), and relative abundance of humans and livestock (ROPD). These data enable analysis of habitat selection and behavioral patterns of medium‐ and large‐bodied mammals. Additionally, we used three anthropogenic disturbances as covariates affecting detection probability, with all factors serving as covariates influencing occupancy rates (Table 1).

**TABLE 1:** Variables used in occupancy modeling.

Covariate	Description	Categories
Psi		
Distance to road	Euclidean distance from camera trap locations to roads extracted using Arcmap 10.8	Disturbance
DTR		
Distance to settlement	Euclidean distance from camera trap locations to settlements extracted using Arcmap 10.8	Disturbance
DTS		
Relative occurrence of people and domestic animals	Ratio of independent detections of humans and domestic animals to total camera working days at each camera trap site	Disturbance
ROPD		
Distance to water	Euclidean distance from camera trap locations to water sources extracted using Arcmap 10.8	Habitat
DTW		
Enhanced vegetation index	Data extracted using Arcmap 10.8 based on data downloaded from Resource and Environment Science and Data Center (https://www.resdc.cn/)	Habitat
EVI		
Slope	Slope at camera trap locations extracted using Arcmap 10.8 based on 30 m DEM data from Geospatial Data Cloud (gscloud.cn)	Habitat
SLP		
Aspect	Aspect at camera trap locations extracted using Arcmap 10.8 based on 30 m DEM data from Geospatial Data Cloud (gscloud.cn)	Habitat
ASP		
Elevation	Elevation based on field surveys	Habitat
ELE		
P		
Distance to road	Euclidean distance from camera trap locations to roads extracted using Arcmap 10.8	Disturbance
DTR		
Distance to settlement	Euclidean distance from camera trap locations to settlements extracted using Arcmap 10.8	Disturbance
DTS		
Relative occurrence of people and domestic animals	Ratio of independent detections of humans and domestic animals to total camera working days at each camera trap site	Disturbance
ROPD		

Elevation was recorded using GPS units at each camera trap location. Slope and aspect were derived from 30 m resolution DEM data obtained from the Geospatial Data Cloud platform of the Computer Network Information Center, Chinese Academy of Sciences (www.gscloud.cn). EVI data were obtained from the MODIS data series regularly released by NASA. Road data were acquired from OpenStreetMap (OSM, https://www.openstreetmap.org). Residential point data were obtained from the National Geomatics Center of China dataset (https://www.ngcc.cn/). Water system data were sourced from the National Geographic Information Resource Directory Service System (www.webmap.cn). Relative abundance of humans and livestock was calculated from independent detections of grazing and human groups. We used ArcToolbox in ArcGIS 10.8 to calculate distances from each camera trap to residential areas, roads, and water sources (Harihar et al. 2014; Cong et al. 2024) (Table 1).

We calculated the variance inflation factor (VIF) to assess multicollinearity among measured variables for all covariates, retaining covariates with VIF < 3 in the model. We further validated this using Pearson correlation coefficients, keeping factors with |*r*| < 0.7. For the retained factors, we constructed models by fitting all possible combinations of covariates. We ranked candidate models using Akaike's information criterion (AIC) and selected models with ΔAIC ≤ 2 as the best models. When multiple optimal models were identified, covariates were determined through model averaging with equal weights. All analyses were conducted using the “unmarked” package in R version 4.3.2 (Wang et al. 2018).

## 3 Results

### 3.1 Monitoring Results

From July 2019 to September 2024, we deployed 62 camera traps, excluding 8 that were damaged or lost, leaving 54 functioning cameras. These cameras accumulated 19,641 working days and captured 25,413 total photographs, including 6204 independent detections. We cataloged all medium‐ and large‐bodied mammals detected in the study area. Among these, we recorded 2646 independent detections of herbivores, 769 independent detections of omnivores, and 281 independent detections of carnivores. Among herbivores, tufted deer had the highest number of independent detections at 1831 (RAI = 9.526), while the large ungulate sambar deer had 199 independent detections (RAI = 1.013). Among carnivores, red fox had the highest number of independent detections at 102 (RAI = 0.519), followed by the large carnivore leopard with 51 independent detections (RAI = 0.260). Among omnivores, wild boar had the highest number of independent detections at 415 (RAI = 2.113), followed by the primate rhesus macaque with 269 independent detections (RAI = 1.370). Additionally, the area experiences significant anthropogenic disturbance, with 722 independent detections of humans and domestic animals (RAI = 2.112), including 148 human activity events and 574 grazing events. Human activities were detected by 80% of camera traps in the area (Table 2).

**TABLE 2:** Camera trap monitoring results, relative abundance index (RAI), and human disturbance events for each wild mammal species in the Kazila Mountain area, 2019–2024.

Diets	Order	Species	Detections	RAI	% site
Mammals					
Herbivore	Artiodactyla	Tufted deer ( *Elaphodus cephalophus* )	1871	9.526	78
Herbivore	Artiodactyla	Chinese serow ( *Capricornis milneedwardsii* )	279	1.420	63
Herbivore	Artiodactyla	Alpine musk deer ( *Moschus chrysogaster* )	254	1.293	44
Herbivore	Artiodactyla	Sambar ( *Rusa unicolor* )	199	1.013	70
Herbivore	Artiodactyla	Dwarf musk deer ( *Moschus berezovskii* )	36	0.183	17
Herbivore	Artiodactyla	Chinese goral ( *Naemorhedus griseus* )	7	0.036	7
Carnivore	Carnivora	Red fox ( *Vulpes vulpes* )	102	0.519	52
Carnivore	Carnivora	Leopard ( *Panthera pardus* )	51	0.260	30
Carnivore	Carnivora	Hog badger ( *Arctonyx collaris* )	29	0.148	30
Carnivore	Carnivora	Lynx ( *Lynx lynx* )	27	0.137	13
Carnivore	Carnivora	Yellow‐throated marten ( *Martes flavigula* )	20	0.102	19
Carnivore	Carnivora	Wolf ( *Canis lupus* )	20	0.102	19
Carnivore	Carnivora	Siberian weasel ( *Mustela sibirica* )	6	0.031	9
Carnivore	Carnivora	Stone marten ( *Martes foina* )	1	0.005	2
Omnivore	Artiodactyla	Wild boar ( *Sus scrofa* )	415	2.113	52
Omnivore	Primates	Rhesus macaque ( *Macaca mulatta* )	269	1.370	57
Omnivore	Primates	Tibetan macaque ( *Macaca thibetana* )	59	0.300	22
Omnivore	Carnivora	Asian black bear ( *Ursus thibetanus* )	25	0.127	20
Omnivore	Carnivora	Brown bear ( *Ursus arctos* )	1	0.005	2
Relative occurrence of humans and domestic animals					
Human activities and grazing			722	2.112	80

### 3.2 Effects of Anthropogenic Disturbance on Temporal Niches of Representative Species

Based on camera trap monitoring results and the Elton Traits database (Mazel et al. 2017), we selected six medium‐ and large‐bodied mammal species for analysis. These included two carnivores: leopard ( *Panthera pardus* ) and red fox ( *Vulpes vulpes* ); two herbivores: sambar ( *Rusa unicolor* ) and tufted deer ( *Elaphodus cephalophus* ); and two omnivores: wild boar ( *Sus scrofa* ) and Rhesus macaque ( *Macaca mulatta* ). These species represent rare wildlife across different trophic levels and ecological niches and are widely distributed across various habitats within the study area.

**FIGURE 2:**
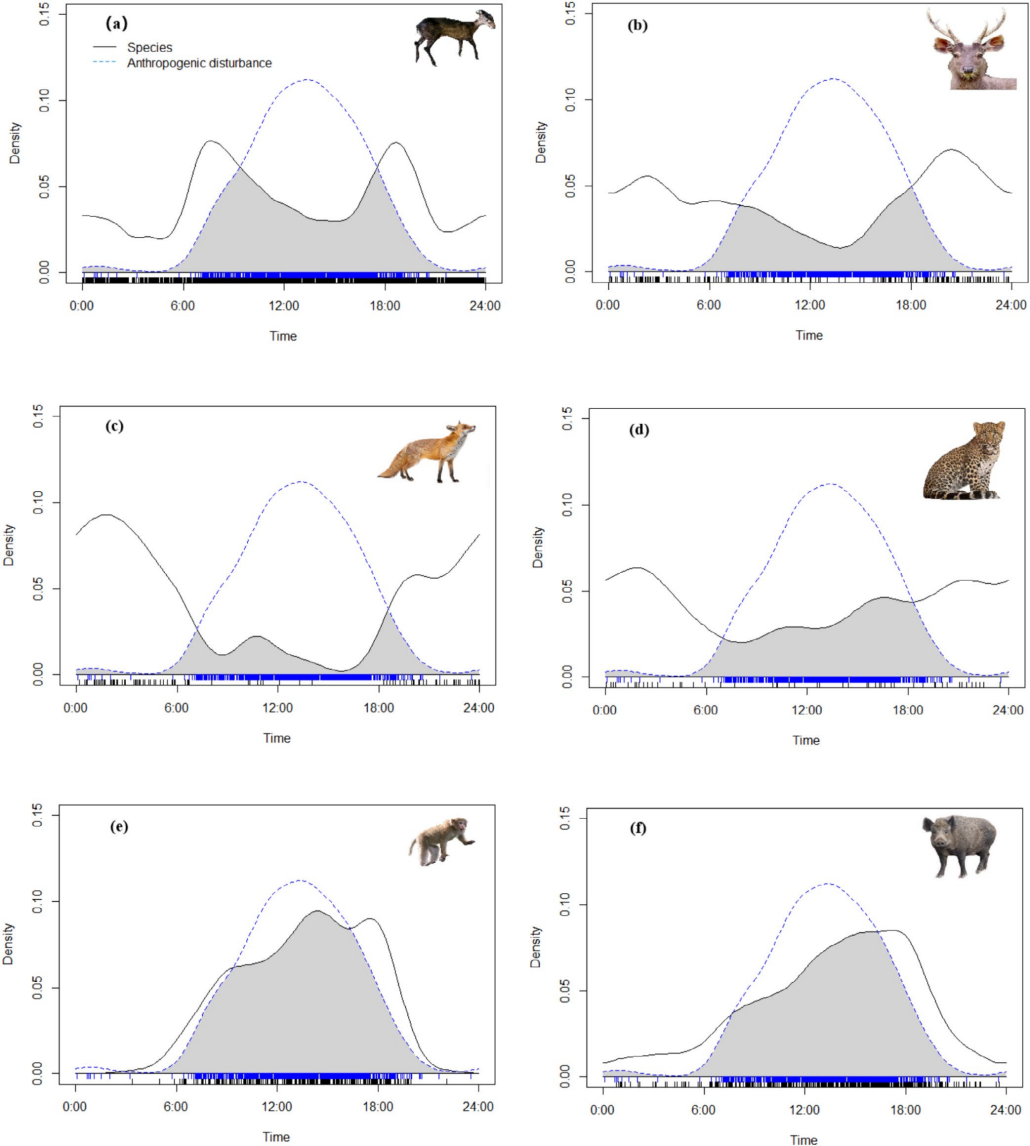
Temporal activity overlap between six representative animal species and human disturbance in the Kazila Mountain area, 2019–2024. (a) Tufted deer, (b) sambar, (c) red fox, (d) leopard, (e) rhesus macaque, (f) wild boar. Blue dashed line: human activities, black solid line: representative species.

Using kernel density estimation, we analyzed activity patterns of leopard, red fox, sambar, tufted deer, wild boar, and Rhesus macaque. Human activities were concentrated during daytime, with intensity increasing from 6:00, peaking at 13:00, then decreasing to near zero around 22:00. Leopards showed activity throughout day and night, with reduced activity from noon to sunset, increasing after sunset, peaking at 2:00, then gradually decreasing. Red foxes were primarily crepuscular, with near‐zero activity from noon to sunset, rapidly increasing after sunset, peaking at 2:00, then decreasing to near zero by 15:00. Sambar maintained consistent activity throughout the day with higher intensity at night, showing two peaks around 3:00 and 21:00. Tufted deer exhibited a bimodal “M‐shaped” pattern, with primary activity during daytime and peaks at 8:00 and 20:00, showing only sporadic activity at night. Wild boars were primarily diurnal, with near‐zero activity at night, increasing from 6:00, peaking around 18:00, then rapidly decreasing to near zero by 22:00. Rhesus macaques were also primarily diurnal, with activity rapidly increasing from 6:00, peaking around 14:00, slightly decreasing, then rising again to a second peak around 18:00 before rapidly decreasing to near zero by 21:00 (Figure 2).

Analysis of diel activity patterns comparing medium‐ and large‐bodied mammals with human activities revealed that leopards showed both diurnal and nocturnal activity, with an overlap index of 0.453 (*p* < 0.001) with human activities, indicating low overlap. Their activity patterns only partially overlapped with human activities during daytime. Red foxes showed the lowest overlap index with human activities at 0.217 (*p* < 0.001), consistent with their nocturnal nature, with minimal overlap occurring only during daytime, primarily at 7:00 (end of morning activity) and 19:00 (start of evening activity). Sambar showed an overlap index of 0.4 (*p* < 0.001) with human disturbance, indicating low overlap and significant temporal partitioning, with partial overlap only during daytime. Tufted deer showed moderate overlap with human activities at 0.557 (*p* < 0.001), as both were primarily diurnal, though tufted deer activity significantly decreased during peak human activity periods. Wild boars showed high overlap with human activities at 0.78 (*p* < 0.001), with no significant temporal partitioning, as both were primarily diurnal. Rhesus macaques showed the highest overlap with human activities at 0.8 (*p* < 0.001), with both showing peak activity during daytime. During dusk, when human activity decreased, macaque activity showed a secondary peak (Figure 2).

### 3.3 Activity Patterns of Representative Species at Sites With Low and High Anthropogenic Disturbance

**FIGURE 3:**
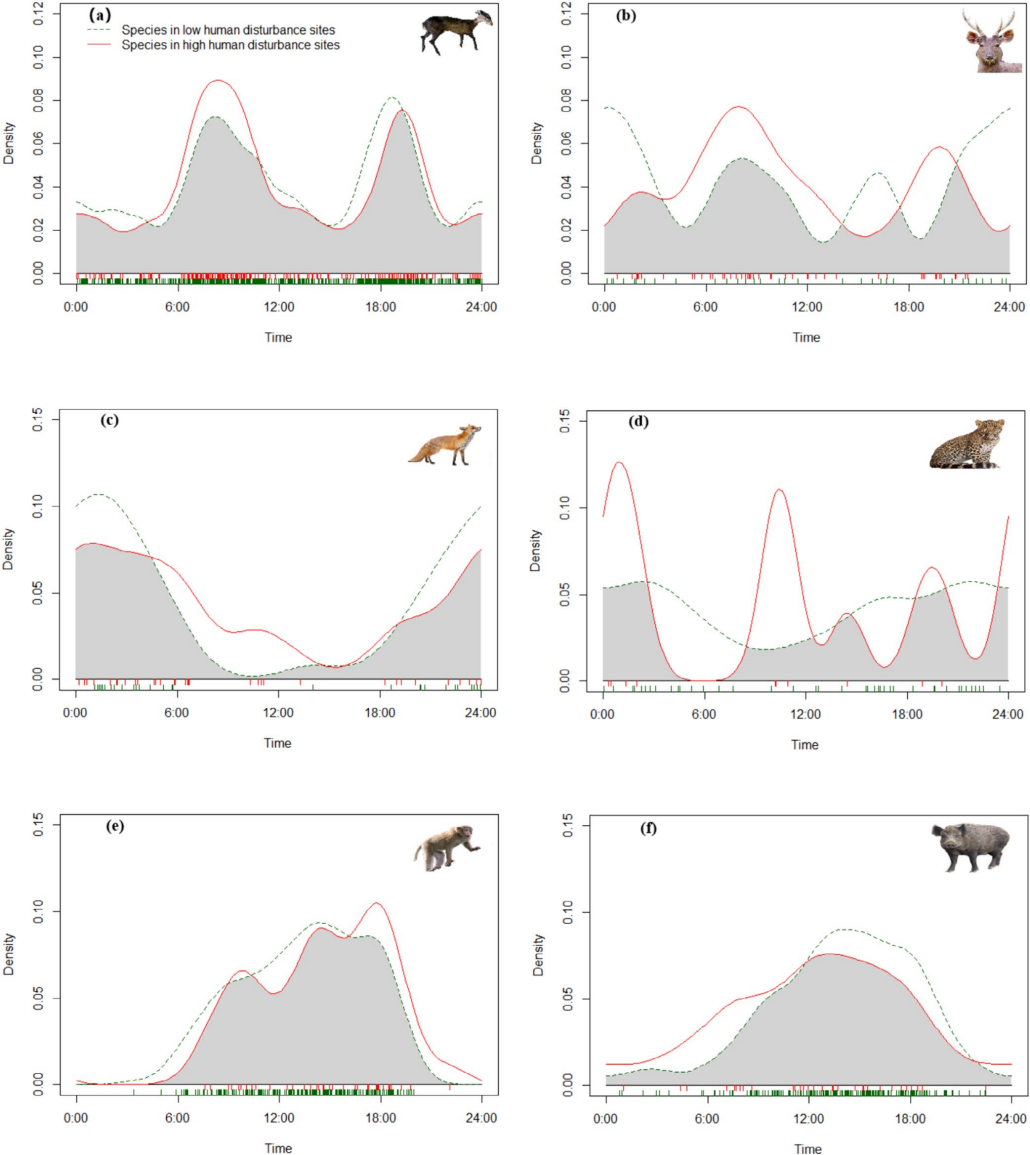
Temporal activity overlap of wild mammals between low and high human disturbance sites in the Kazila Mountain area, 2019–2024. (a) Tufted deer, (b) sambar, (c) red fox, (d) leopard, (e) rhesus macaque, (f) wild boar. Green dashed line: low human disturbance sites, red solid line: high human disturbance sites.

Analysis of activity patterns for the six species in areas with low and high human activity levels revealed differences in their activity patterns across these regions. Leopards showed significant differences in activity patterns between the two areas, with an overlap coefficient of 0.541 (*p* = 0.01). Only 10 detections were recorded in high human activity areas compared to 41 in low disturbance areas. While other species showed no significant differences, their activity patterns exhibited some modifications. Sambar showed two activity peaks in high human activity areas, but three peaks in low human activity areas. Rhesus macaques showed delayed activity peaks around 18:00 in high human activity areas compared to peaks around 14:00 in low human activity areas. Red foxes, tufted deer, and wild boars showed minimal changes in their activity rhythms across different human activity levels (Figure 3).

### 3.4 Effects of Anthropogenic Disturbance and Environmental Variables on Spatial Niches of Representative Species

Based on occupancy model results (ΔAIC ≤ 2; Table 3), leopards showed a detection probability of 0.186 and an occupancy rate of 0.337, the lowest predicted occupancy among all species. Red foxes showed a detection probability of 0.134 and an actual occupancy rate of 0.889, the highest among species. Sambar showed a detection probability of 0.22 and an occupancy rate of 0.674. Tufted deer showed the highest detection probability (0.566) and an occupancy rate of 0.637. Rhesus macaques showed a detection probability of 0.209 and an occupancy rate of 0.558. Wild boars showed a detection probability of 0.234 and an occupancy rate of 0.548.

**TABLE 3:** Summary of occupancy rate and detection probability of different species for the optimal models (ΔAIC ≤ 2).

Species	Model	*K*	AIC	ΔAIC	AIC weight	*ψ*	*p*
Leopard	Psi(DTS); P(EVI + DTS + ROPD)	6	136.737	0.000	0.156	0.441	0.185
	Psi(DTS + ROPD); P(EVI + DTS + ROPD)	7	137.046	0.310	0.133	0.377	0.2
	Psi(DTS); P(DTS)	4	137.179	0.442	0.125	0.285	0.173
	Psi(DTS); P(DTS + DTR)	5	137.693	0.956	0.096	0.269	0.171
	Psi(DTS + ROPD); P(DTS + ROPD)	6	137.694	0.958	0.096	0.313	0.196
	Psi(DTS); P(DTS+ ROPD)	5	138.046	1.309	0.081	0.388	0.183
	Psi(DTS + ROPD); P(DTS + DTR + ROPD)	7	138.291	1.554	0.072	0.297	0.197
	Psi(DTS + ROPD); P(DTS + DTR)	6	138.354	1.617	0.069	0.264	0.189
	Psi(DTS + ROPD); P(DTS)	5	138.363	1.627	0.069	0.278	0.187
	Psi(DTR + DTS + ROPD); P(EVI + DTR + ROPD)	7	138.385	1.648	0.062	0.455	0.176
	Psi(DTR + DTS); P(EVI + DTS + ROPD)	6	138.584	1.847	0.041	0.441	0.185
	Model average					0.337	0.186
Red fox	Psi(DTR + DTS + ROPD); P(DTR + ROPD)	7	308.17	0	0.568	0.889	0.135
	Psi(DTR + DTS + ROPD); P(DTR + DTW + ROPD)	8	310.078	1.909	0.219	0.890	0.134
	Psi(DTR + DTS + ROPD); P(ELE + DTR + ROPD)	8	310.121	1.951	0.214	0.887	0.132
	Model average					0.889	0.134
Sambar	Psi(ROPD); P(DTS + ROPD)	5	406.823	0	0.143	0.682	0.216
	Psi(DTR + ROPD); P(DTS + ROPD)	6	407.18	0.358	0.12	0.68	0.217
	Psi(ROPD); P(DTR + DTS + ROPD)	6	407.461	0.638	0.104	0.679	0.221
	Psi(DTR + ROPD); P(DTR + DTS + ROPD)	7	407.671	0.848	0.094	0.677	0.222
	Psi(DTR + DTW + ROPD); P(DTS + ROPD)	7	408.247	1.424	0.07	0.673	0.219
	Psi(DTS + ROPD); P(DTS + ROPD)	6	408.326	1.503	0.067	0.677	0.217
	Psi(SLP + ROPD); P(DTS + ROPD)	6	408.514	1.691	0.061	0.681	0.216
	Psi(DTS + DTR + ROPD); P(DTS + ROPD)	7	408.556	1.734	0.06	0.676	0.217
	Psi(EVI + ROPD); P(DTS + ROPD)	6	408.586	1.763	0.059	0.682	0.217
	Psi(SLP + DTR + ROPD); P(DTS + ROPD)	7	408.623	1.8	0.058	0.682	0.216
	Psi(DTR + DTW + ROPD); P(DTR + DTRP + ROPD)	8	408.672	1.849	0.057	0.672	0.224
	Psi(ELE + ROPD); P(DTS + ROPD)	6	408.783	1.96	0.054	0.683	0.216
	Psi(DTW + ROPD); P(DTS + ROPD)	6	408.813	1.991	0.053	0.681	0.216
	Model average					0.674	0.22
Tufted deer	Psi(ELE); P(DTR)	4	558.332	0	0.109	0.641	0.566
	Psi(ELE); P(DTR + ROPD)	5	558.517	0.185	0.099	0.638	0.563
	Psi(ELE); P(DTR + DTRP)	5	559.114	0.782	0.074	0.641	0.569
	Psi(SLP + ELE); P(DTR)	5	559.327	0.995	0.066	0.642	0.566
	Psi(ELE); P(DTR + DTRP + ROPD)	6	559.37	1.038	0.065	0.638	0.566
	Psi(ELE + ROPD); P(DTR)	5	559.4	1.068	0.064	0.645	0.565
	Psi(SLP + ELE); P(DTR + ROPD)	6	559.504	1.172	0.061	0.64	0.563

	Psi(ELE + ROPD); P(DTR + ROPD)	6	559.661	1.328	0.056	0.641	0.563
	Psi(ELE + DTRP); P(DTR)	5	559.913	1.581	0.049	0.642	0.566
	Psi(ELE + DTW); P(DTR)	5	559.921	1.589	0.049	0.64	0.566
	Psi(ELE + DTW); P(DTR + ROPD)	6	560.055	1.723	0.046	0.637	0.564
	Psi(SLP + ELE + ROPD); P(DTR)	6	560.079	1.747	0.045	0.647	0.565
	Psi(ELE + DTRP); P(DTR + ROPD)	6	560.095	1.763	0.045	0.638	0.563
	Psi(SLP + ELE); P(DTR + DTRP)	6	560.156	1.823	0.044	0.642	0.569
	Psi(ELE + ROPD); P(DTR + DTRP)	6	560.163	1.83	0.044	0.645	0.568
	Psi(EVI + ELE); P(DTR)	5	560.164	1.832	0.044	0.642	0.566
	Psi(ELE + DTR); P(DTR)	5	560.331	1.999	0.04	0.641	0.566
Model average					0.637	0.566
Rhesus macaque	Psi(DTR + ROPD); P(EVI + DTRP + ROPD)	7	315.208	0	0.387	0.556	0.212
	Psi(DTR); P(EVI + DTRP + ROPD)	6	315.678	0.47	0.306	0.566	0.196
	Psi(DTR + ROPD); P(DTS + ROPD)	6	316.972	1.764	0.16	0.554	0.212
	Psi(DTR + DTS + ROPD); P(EVI + DTS + ROPD)	8	317.163	1.955	0.146	0.556	0.214
	Model average					0.558	0.209
Wild boar	Psi(DTR + ROPD); P(EVI + DTRP + ROPD)	7	333.829	0	0.16	0.611	0.23
	Psi(DTR + DTRP + ROPD); P(EVI)	6	333.972	0.143	0.149	0.529	0.232
	Psi(DTRP + ROPD); P(EVI)	5	334.779	0.95	0.099	0.516	0.236
	Psi(DTR + DTS + ROPD); P(SLP + EVI)	7	335.021	1.193	0.088	0.521	0.234
	Psi(DTR + DTS + ROPD); P(EVI + ROPD)	7	335.022	1.193	0.088	0.544	0.233
	Psi(DTR + ROPD); P(SLP + EVI + ROPD)	7	335.139	1.31	0.083	0.601	0.233
	Psi(DTR + ROPD); P(EVI)	5	335.306	1.478	0.076	0.519	0.244
	Psi(DTR + DTRP + ROPD); P(EVI + ELE)	7	335.369	1.54	0.074	0.532	0.231
	Psi(DTR + DTRP + ROPD); P(EVI + DTRP + ROPD)	8	335.66	1.832	0.064	0.609	0.227
	Psi(DTS + ROPD); P(SLP + EVI)	6	335.781	1.952	0.06	0.513	0.237
	Psi(DTRP + ROPD); P(EVI + DTR)	6	335.795	1.966	0.06	0.531	0.234
	Model average					0.548	0.234

**FIGURE 4:**
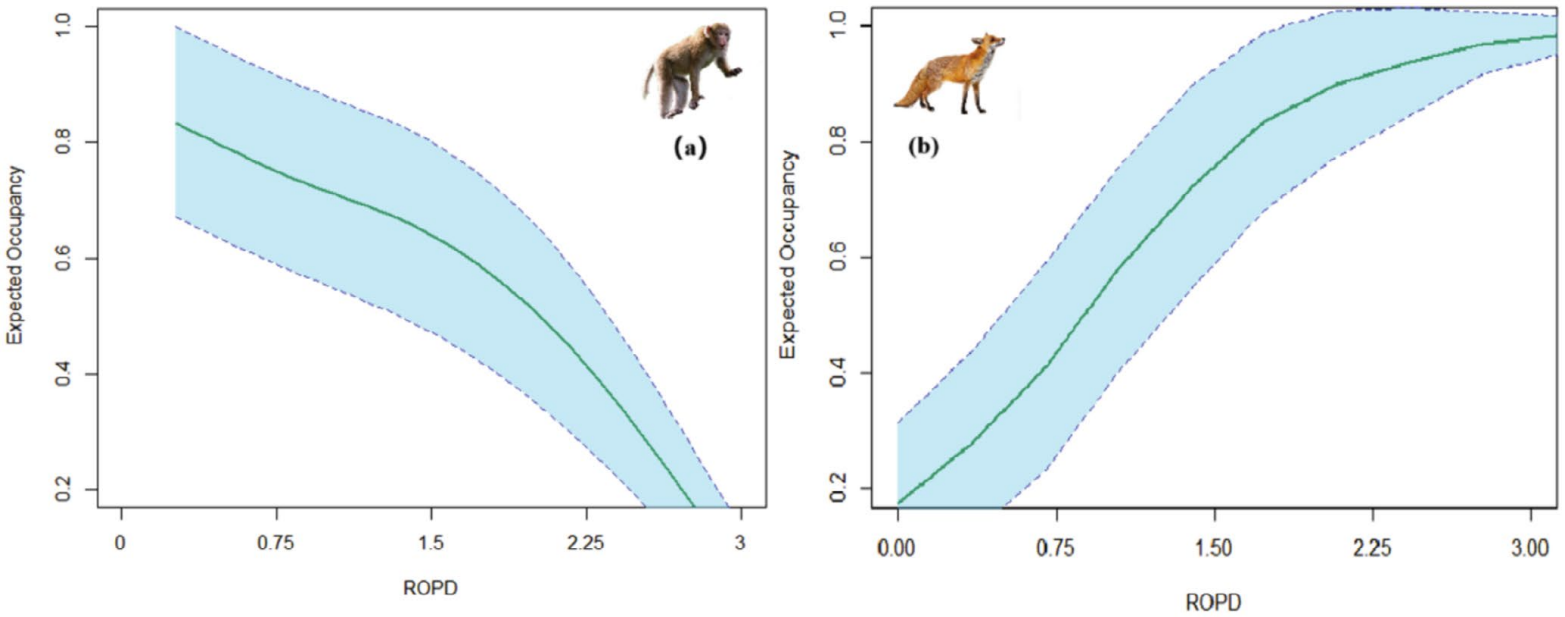
Relationships between occupancy rates of representative species and anthropogenic disturbance variables under the best models (AIC ≤ 2). (a) Rhesus macaque, (b) red fox. Solid lines represent fitted polynomial regression, and blue shaded areas represent 95% confidence intervals.

Based on species occupancy results (Table 4), environmental variables significantly affected tufted deer occupancy, while red fox and rhesus macaque occupancy were significantly influenced by anthropogenic disturbance, with no significant effects on other species. Tufted deer showed sensitivity to elevation (*β* = 0.736, *p* = 0.047), with occupancy increasing with elevation. Relative occurrence of humans and domestic animals positively influenced red fox occupancy (*β* = 0.788, *p* = 0.013) (Figure 4), with occupancy increasing with higher relative occurrence. Rhesus macaque occupancy was negatively affected by relative occurrence of humans and domestic animals (*β* = −0.994, *p* = 0.043) (Figure 4), with occupancy decreasing as relative occurrence increased. Leopard occupancy showed positive correlations with distance to settlement, distance to roads, and relative occurrence of humans and domestic animals, indicating preference for areas with less human disturbance, though grazing may provide some attraction. Red fox occupancy also showed positive correlation with distance to roads and negative correlation with distance to water sources. Both herbivores (sambar and tufted deer) showed negative correlations with distance to roads and settlements, while preferring areas with gentler slopes and higher enhanced vegetation index (EVI). Both omnivores (wild boar and rhesus macaque) preferred areas with higher EVI. Additionally, wild boar showed negative correlations with distance to roads and settlements, and positive correlation with relative occurrence of humans and domestic animals, suggesting potential attraction to human‐disturbed areas.

**TABLE 4:** Covariates influencing occupancy and detection probabilities of representative species based on the best models (ΔAIC ≤ 2).

Species	Model	Covariates	Estimate	Standard error	*z*	*p*
Leopard	Occupancy	Int	−0.837	0.792	0.939	0.348
		EVI	−0.506	1.035	0.488	0.625
		DTRP	1.873	1.795	1.043	0.297
		DTR	0.138	0.341	0.405	0.686
		ROPD	1.036	0.440	0.719	0.472
	Detection	Int	−1.800	0.355	5.062	< 0.01**
		DTS	−0.988	0.558	1.771	0.077
		ROPD	−0.166	0.309	0.539	0.590
		DTR	0.042	0.055	0.077	0.939
Red fox	Occupancy	Int	0.217	0.193	0.612	0.540
		EVI	1.298	0.958	0.663	0.507
		DTR	−0.222	0.995	0.544	0.587
		DTW	−0.224	0.115	0.042	0.966
		ROPD	0.788	0.522	2.495	0.013*
		SLP	−0.118	0.131	0.356	0.722
	Detection	Int	−1.985	0.265	7.487	< 0.01**
		DTRP	0.225	0.126	0.996	0.319
		ROPD	−0.129	0.290	0.446	0.656
		DTR	0.671	0.352	1.905	0.057
Sambar	Occupancy	Int	4.891	2.097	2.333	0.02*
		DTR	0.136	0.366	0.574	0.566
		ROPD	0.370	0.694	0.533	0.594
		DTS	−0.459	0.219	0.21	0.834
		SLP	−0.839	0.217	0.178	0.859
		EVI	0.183	0.167	0.11	0.913
		ELE	0.055	0.119	0.046	0.963
		DTW	0.506	0.260	0.195	0.845
	Detection	Int	−1.349	0.143	9.447	< 0.01**
		DTRP	−0.293	0.128	2.278	0.023*
		ROPD	−0.403	0.161	2.505	0.012*
		DTR	−0.034	0.090	0.376	0.707
Tufted deer	Occupancy	Int	0.598	0.319	1.877	0.061
		ELE	0.737	0.402	1.834	0.047*
		SLP	−0.666	0.202	0.33	0.742
		ROPD	−0.559	0.176	0.318	0.750
		DTRP	−0.192	0.116	0.165	0.869
		DTW	0.198	0.118	0.167	0.867
		EVI	0.059	0.072	0.082	0.935
		DTR	−0.045	0.071	0.006	0.995

	Detection	Int	0.268	0.110	2.438	0.015*
		DTR	−0.421	0.129	3.26	0.001**
		ROPD	−0.070	0.137	0.515	0.607
DTRP		−0.025	0.069	0.353	0.724
Rhesus macaque	Occupancy	Int	1.198	0.740	1.509	0.131
		EVI	0.731	0.637	1.149	0.251
		ROPD	−0.994	0.534	2.001	0.043*
		DTRP	3.094	1.546	1.362	0.183
	Detection	Int	−1.414	0.178	7.948	< 0.01**
		DTR	−0.421	0.169	2.482	0.013**
		ROPD	−0.153	0.175	0.870	0.384
		DTRP	0.030	0.047	0.062	0.951
Wild boar	Occupancy	Int	0.653	1.227	0.532	0.594
		EVI	1.049	0.758	1.384	0.166
		DTS	−0.195	0.533	0.365	0.715
		ROPD	1.106	0.297	0.481	0.630
		SLP	−0.107	0.311	0.345	0.730
		DTR	−0.26	0.140	0.184	0.854
		ELE	0.18	0.127	0.145	0.885
	Detection	Int	−1.318	0.179	7.386	< 0.01**
		DTR	−0.300	0.259	1.158	0.247
		DTRP	−0.276	0.261	1.057	0.291
		ROPD	−0.493	0.229	2.152	0.031*

*Note:* The different superscript letters represent significance, **p < 0.01; *0.01 < *p* < 0.05.

Analysis of species detection probabilities (Table 4) revealed that sambar showed significant negative correlations with distance to settlement, distance to roads, and relative abundance of humans and livestock, indicating that grazing and human activities influence their detection probability. Tufted deer detection probability also showed a significant negative correlation with distance to roads, suggesting potential attraction to human‐modified landscapes for ungulate species. Wild boar detection probability showed a significant negative correlation with relative abundance of humans and livestock, indicating avoidance of areas with high real‐time human activity. Rhesus macaque detection probability showed a significant negative correlation with distance to roads. While leopard and red fox detection probabilities showed no significant effects, both showed negative correlations with relative abundance of humans and livestock.

## 4 Discussion

Camera trap technology is commonly applied in wildlife resource surveys within nature reserves (Liu et al. 2018). Through statistical analysis of independent detections and capture times across different species, it provides a scientific basis for biodiversity assessment and species conservation in study areas (Sheng et al. 2014). With the increasing sophistication of camera trap monitoring, more researchers are applying it to study animal responses to anthropogenic disturbances, including real‐time activities like grazing and mushroom harvesting, as well as long‐term disturbances like construction and road development (Li, Hu, et al. 2022; Dirzo et al. 2014). Through intensive camera trap monitoring, we investigated wildlife responses to various anthropogenic disturbances in the mountainous regions of southwest China. From July 2019 to September 2024, the most frequently detected group in the Kazila Mountain area was ungulates, including tufted deer, wild boar, Chinese serow, and alpine musk deer; followed by primates, with carnivores being the least detected (Table 2). The community structure composed of multiple primary consumers and some secondary consumers is considered beneficial for stabilizing food chains and webs, as well as maintaining ecosystem balance (Li, Zhang, et al. 2024). Additionally, human disturbances such as grazing accounted for 80% of detections, indicating frequent human activity in this region.

Activity rhythms represent evolutionary adaptations to environmental changes, reflecting species' responses to external conditions (Gaynor et al. 2018). These rhythms are influenced by food resources, social structures, and human modifications and disturbances. Over time, natural selection continuously shapes species' activity patterns, with anthropogenic disturbances playing a significant role in this process (Li, Zhang, et al. 2024; Li, Bleisch, et al. 2024). Our results show that leopards concentrate their activity during crepuscular periods, consistent with findings by Li, Zhang, et al. (2024) and Liu et al. (2021) in the Northeast Tiger and Leopard National Park. Red fox diel activity patterns align with Dong Wang et al. (2022) in the Yangtze River source region, showing peak activity before sunrise and after sunset, with almost no activity during daytime, indicating clear nocturnal behavior. Both ungulate species, sambar and tufted deer, exhibit bimodal activity patterns primarily during crepuscular periods, consistent with Li, Zhang, et al. (2024) in southwest China's mountainous regions. Except for wild boar, all ungulates primarily show activity during daytime and dusk, with significant activity peaks around sunrise and sunset, consistent with our findings (Li, Xue, et al. 2022). Rhesus macaques concentrate activity during daytime; though in our study, their two activity peaks occurred closer to sunset, differing from Li, Xue, et al. (2022).

In response to anthropogenic disturbances, animals may exhibit various behavioral responses, with activity rhythm adjustments being one of the most important (Xu et al. 2021). The plasticity of wildlife activity patterns in the temporal dimension helps them avoid human disturbances and achieve coexistence, a phenomenon also observed in other studies (Hu et al. 2022; Shi et al. 2020). In our study area, where human activities are concentrated during daytime, different species groups have adopted distinct behavioral strategies to avoid human presence. Specifically, species modify their diel activity patterns to avoid core human activity periods or peaks, thereby altering their temporal overlap with human activities (Gaynor et al. 2018). Our results show significant differences in diel activity intensity between all species and human disturbances, with varying degrees of temporal partitioning among different groups. Herbivores (sambar, tufted deer) and carnivores (leopard, red fox) show high temporal partitioning with human disturbance, with moderate overlap only for tufted deer and low overlap for others. This aligns with previous findings that herbivores and carnivores are susceptible to disturbance competition and grazing intensity/quality (Schieltz and Rubenstein 2016) and that large herbivores and carnivores may modify daily activity patterns to reduce conflicts with human grazing activities (Xu et al. 2024). Both omnivorous species show high overlap with human disturbance but significant differences in activity patterns, explained by subtle differences in peak activity timing (Petridou et al. 2023). For instance, wild boars show slightly later evening activity peaks than human disturbance peaks, with higher post‐sunset activity levels than human disturbance. Both omnivores' activity patterns align with Li et al. (2024) in the Northeast Tiger and Leopard National Park, showing increased daytime activity with higher human presence. Field‐recorded animal activity patterns likely represent a balance between energy acquisition and human avoidance (Kautz et al. 2022; Frid and Dill 2001). Our results show that all species groups exhibit activity patterns that avoid human disturbance peaks, indicating that wildlife diel rhythms are indeed influenced by human disturbance and show different patterns under varying disturbance intensities.

Wildlife population spatial distribution is influenced by multiple abiotic and biotic factors (Hu et al. 2022). Through long‐term monitoring and comprehensive analysis, this study provides the first explanation of occupancy patterns and influencing factors for three representative groups in the Kazila Mountain ecosystem. The two carnivores show partial differences in occupancy patterns: Leopard occupancy decreases with higher Enhanced Vegetation Index (EVI), as more open areas facilitate hunting for large carnivores (Wang et al. 2018), while red foxes prefer areas with higher EVI, likely due to their broader diet including birds, invertebrates, and rodents (Rossa et al. 2021). Additionally, red foxes select areas with gentler slopes and closer proximity to water sources, reflecting movement strategies that maximize energy efficiency (Gaudry et al. 2015). Both herbivores show consistent occupancy patterns, with occupancy increasing with elevation, EVI, and distance to water sources, aligning with previous research (Hu et al. 2018; Zhang et al. 2011). Herbivores prefer high‐altitude areas where coniferous forests intermingle with subalpine shrubs and meadows, providing abundant food resources (Li, Zhang, et al. 2024), and select gentler slopes that facilitate foraging and slow movement, reducing overall activity and energy expenditure (Li, Xue, et al. 2022). Both omnivores, primarily herbivorous but also consuming animal matter, show increased occupancy with higher EVI, as these areas not only provide abundant plant resources but also support diverse insect populations (Wei et al. 2010; Li, Xue, et al. 2022). Wild boars share similar habitat preferences with other ungulates, favoring high‐altitude areas with gentler slopes (Guojing et al. 2019).

The impact of anthropogenic disturbance on wildlife spatial distribution depends on disturbance type and intensity, and typically varies by species (Gaynor et al. 2018; Laméris et al. 2019). Human activities may reduce vegetation heterogeneity and decrease species diversity and richness in study areas (Evans et al. 2015). Our results show different effects of human disturbance on three representative species. Both carnivores show increased occupancy with higher relative abundance of humans and livestock, likely because livestock serve as alternative prey sources for medium and large carnivores like leopards and red foxes (Trainor and Schmitz 2014), with numerous reports of livestock predation by large carnivores in previous studies (Alexander et al. 2015). Leopards prefer areas farther from human disturbance, consistent with findings by Li et al. (2024c) and Wang et al. (2018) in the Northeast Tiger and Leopard National Park. Red foxes prefer areas closer to roads, aligning with previous research (Lazzeri et al. 2024; Chen et al. 2023), suggesting they seek proximity to human settlements as refuge from larger predators (Berger 2007), though some studies attribute this to abundant rodent populations near human activity areas providing favorable foraging grounds (Lazzeri et al. 2024). This may also be related to the red fox's preference for foraging on young livestock individuals (Hacker et al. 2022; Li, Bleisch, et al. 2024). While disturbances like logging and grazing provide some refuge for herbivores, published studies show medium and large herbivores are susceptible to human activity and livestock density (Feng et al. 2021; Schieltz and Rubenstein 2016). Our results align with this, showing tufted deer occupancy decreases with higher relative abundance of humans and livestock, while sambar prefer areas farther from roads. Among omnivores, wild boar occupancy decreases with greater distance to roads and settlements, consistent with findings in northern Greece (Petridou et al. 2023), indicating higher activity in human‐disturbed areas likely seeking food resources (Rutten et al. 2019). In contrast, rhesus macaques show opposite responses, selecting areas farther from roads with lower relative abundance of humans and livestock, consistent with previous research in Sichuan's Baihe Nature Reserve (Li et al. 2015), where primates' high vigilance leads them to prefer well‐concealed habitats to avoid disturbance.

## 5 Conclusion

In summary, this study examined species across different trophic levels to compare activity rhythms and assess temporal responses to human activities. Leopards and red foxes were selected as representatives of apex and mesopredators, respectively; sambar and tufted deer as typical herbivores; and wild boars and rhesus macaques as generalist omnivores. By analyzing diel activity patterns, applying occupancy models, and evaluating anthropogenic impacts on these three functional groups in the Kazila Mountain region, we found that their activity patterns are influenced by human disturbance and exhibit a degree of behavioral plasticity. Different species adjusted their spatiotemporal activity in response to human presence, aligning with findings from other studies on wildlife adaptation to anthropogenic pressures (Greenberg and Holekamp 2017; Reilly et al. 2017). Model parameters should be interpreted as predictive associations; underlying mechanisms require further validation through experimental or long‐term observational studies.

Although the magnitude of human disturbance effects varies across groups and remains somewhat uncertain, our results underscore the importance of incorporating strategies to mitigate anthropogenic impacts into conservation planning for medium‐ and large‐sized mammals. Notably, many disturbances observed during this study originated from domestic animals such as yaks, sheep, and dogs. Previous research has shown that livestock grazing (Zhang et al. 2017) and the presence of domestic dogs (Hughes and Macdonald 2013; Shi et al. 2020) negatively influence the behavior of rare wildlife. Consequently, conservation efforts should aim to strike a balance between local economic development, the livelihood needs of residents, and the protection of wildlife by minimizing frequent human disturbances in natural habitats.

## Author Contributions

**Qiuxian Li:** conceptualization (lead), data curation (equal), formal analysis (lead), investigation (equal), methodology (lead), resources (equal), writing – original draft (equal), writing – review and editing (equal). **Xinyu Cui:** formal analysis (supporting), investigation (equal), resources (equal). **Qingsong Jiang:** investigation (equal), resources (equal). **Hangshu Xiao:** investigation (equal), resources (equal). **Assan Meshach:** investigation (equal), resources (equal). **Huaqiang Zhou:** investigation (equal), resources (equal). **Yin Li:** investigation (lead), resources (equal). **Zejun Zhang:** funding acquisition (equal), project administration (equal). **Mingsheng Hong:** funding acquisition (equal), project administration (equal), supervision (equal), writing – review and editing (equal).

## Conflicts of Interest

The authors declare no conflicts of interest.

## Data Availability

The original contributions presented in this study are included in the article; further inquiries can be directed to the corresponding author.
